# The individualistic fallacy, ecological studies and instrumental variables: a causal interpretation

**DOI:** 10.1186/1742-7622-11-18

**Published:** 2014-11-19

**Authors:** Tom Loney, Nico J Nagelkerke

**Affiliations:** Institute of Public Health, College of Medicine and Health Sciences, United Arab Emirates University, Al Ain, United Arab Emirates

**Keywords:** Aggregate studies, Bias, Ecological fallacy, Environmental health, Instrumental variables, Methodological individualism

## Abstract

The validity of ecological studies in epidemiology for inferring causal relationships has been widely challenged as observed associations could be biased by the Ecological Fallacy. We reconsider the important design components of ecological studies, and discuss the conditions that may lead to spurious associations. Ecological associations are useful and valid when the ecological exposures can be interpreted as Instrumental Variables. A suitable example may be a time series analysis of environmental pollution (e.g. particulate matter with an aerodynamic diameter of <10 micrometres; PM_10_) and health outcomes (e.g. hospital admissions for acute myocardial infarction) as environmental pollution levels are a cause of individual exposure levels and not just an aggregate measurement. Ecological exposures may also be employed in situations (perhaps rare) where individual exposures are known but their associations with health outcomes are confounded by unknown or unquantifiable factors. Ecological associations have a notorious reputation in epidemiology and individualistic associations are considered superior to ecological associations because of the “ecological fallacy”. We have argued that this is incorrect in situations in which ecological or aggregate exposures can serve as an instrumental variable and associations between individual exposure and outcome are likely to be confounded by unmeasured variables.

## Introduction

Ecological studies are epidemiological investigations in which either the units of analysis are populations or groups of people, as opposed to individuals, or exposures are only known at the population level while outcomes may be known at the individual level. Specifically, ecological variables are properties of groups, organisations, or places, whereas individual-level variables are properties of each person [1]. Generally, public and environmental health researchers utilise ecological study designs to explore potential causal associations between one or more exposures and a specific health outcome when alternative study designs (e.g. case–control, cohort, randomised controlled trial) are not possible or relevant. For example, an ecologic study is the most appropriate research design if we were interested in the effect of a macro-level governmental policy change, such as an inner-city traffic congestion charge to reduce carbon emissions and improve air quality, on a particular aggregate health outcome (e.g. number of consultations for childhood respiratory disorders).

### Ecological fallacy

An ecological study design may also be utilised when the underlying question regards individuals, such as when one is interested in the effects of air quality on health. In this context, ecological studies are potentially susceptible to the “ecological fallacy”; biases that may occur when an observed relationship between aggregated variables differs from the true, i.e. causal, association at an individual level [2]. Indeed, since Robinson in 1950 showed that correlations on an individual level can differ markedly from those on an aggregate, ecological level, epidemiological textbooks, papers and courses have warned us about the dangers of this ecological fallacy [3, 4].

Although ecological studies are still widely carried out, this sceptical attitude towards ecological studies has led to the “individualistic primacy”, i.e. the belief that associations on an individual level are intrinsically more truthful, i.e. better reflecting causal relationships, than those on an ecological level [5]. Public and environmental health practitioners in particular tend to assume low level validity of ecological studies. An additional reason for this scepticism may be that the implicit objective of mainstream epidemiological research is the understanding of disease aetiology, which is intrinsically an individualistic process. For example, asbestos exposure in a neighbourhood can only cause disease in individuals who were themselves exposed.

The supposed (ab)use of ecological associations has even given rise to some occasional mockery (spoof), for instance by showing an association between Nobel prizes awarded to a country, as a proxy for cognitive functioning, and per capita chocolate consumption [6]. This rather facetious example illustrates the potential weaknesses of ecological studies. First, of course, there are many obvious confounders that may explain the association between aggregate chocolate consumption and the number of Nobel prizes in a country, but also – more seriously, one can doubt whether aggregate consumption can be considered a good predictor or “cause” of individual chocolate consumption (by the Nobel laureates). Although originally formulated with nuance, the Simpson’s “paradox” which has also become part of the canon of epidemiology, has strengthened this attitude [7]. Simpson’s paradox (or the Yule-Simpson effect) is a relationship between two variables that is observed in different groups of data that reverses when these groups are combined or aggregated. That said, Rothman [8] posited that Simpson’s paradox is not a ‘true’ paradox, rather the logical consequence of failing to recognise the presence of confounding variables. In the above example, it might be that academics tend to have the lowest chocolate consumption of all citizens, so that within a country the association is the inverse of that on a country level.

The concept of ecological fallacy, however, seems to encompass several, different, potential biases, which may explain why many authors seem to have difficulty precisely delineating this concept. One of these biases, as explained above, is classical confounding, i.e. that ecological, aggregated, units differ in more (unmeasured) aspects than the (aggregate) exposure of interest, and that these aspects may be related to the outcome. This ecological confounding may also be caused by confounding on an individual level (say by age or sex) when those confounders cannot be measured or observed and their distribution differs among ecological units. Another form of bias is model specification bias, which can occur when the relationship between individual exposure and outcome is non-linear and the mean outcome and the outcome of the mean exposure therefore differ [9]. The resulting difference between the expectation of an estimator from an ecological study and the individual-level parameter of interest is also known as ecological or cross-level bias. In general, this latter form of bias is unlikely to introduce associations that do not exist, but rather leads to biased estimates in the correct direction of effects, which is often less troubling than the possibility of introducing spurious associations and thus qualitatively incorrect conclusions. Tests for the null hypothesis of no associations are still unbiased. The same is true for the apparent difference in association that can occur due to the process of aggregation itself. The correlation between individual income and health expenditure on an individual level and that of *average* income and *average* health expenditure on a municipal level is bound to differ, either magnified or diluted, even when the latter association is completely explained by the former. In that case however, conclusions would still hold qualitatively as the direction of the association will be the same. As such, it is qualitative error, i.e. reversion of the association direction due to aggregation, or the introduction of spurious effects, that is often of greatest concern in epidemiology and is the main issue we consider in this paper.

### Individualistic fallacy

Although this negative attitude towards ecological studies has been criticised for putting too much emphasis on the individual as the unit for analysis [10], which even led to the term “individualistic fallacy”, this has gained much less attention in the epidemiological literature. Some of this critique pointed at the intrinsic, conceptual difference between variables on an individual and group level [2]. For example, that in addition to personal poverty (say) the poverty or deprivation of the society or area in which one lives also matters. In addition, some variables only exist, or make sense, at an aggregated or societal level, such as whether one lives in a democracy or not. In other contexts, societal level variables also obviously impact relevantly on an individual’s risk. For example, the risk of contracting a sexually transmitted infection is not only determined by an individual’s risk behaviour but also by the population prevalence of the infection, which in turn is largely determined by “societal” factors such as the connectedness of sexual networks. While this criticism of the individual primacy is correct, we feel that this critique overlooks some important points. Here we argue that even when the emphasis is clearly on the individual, with the ultimate objective of understanding the aetiology of disease, ecological analyses can be more “truthful” than individualistic analyses and may avoid a large portion of confounding on the individual level.

## Objectives

The aim of this paper is to extricate the confusion surrounding ecological studies by re-evaluating the important design components and conditions that may lead to bias. We propose that ecological associations are useful and informative for individual associations when the ecological exposures can be interpreted as Instrumental Variables (IV).

## Instrumental variables

**Figure 1:**
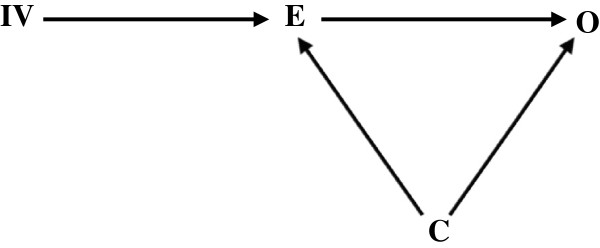
**Markov graph showing the effects of individual exposures (E) on their health outcome (O) in the context of observational studies.** Both E and O can be affected by other variables, including (unknown) confounders (C). The existence of an Instrumental variable (IV) makes it possible to establish a causal relationship between E and O.

Public health epidemiology cannot lay claim to discovering the utility of IV in ecological studies as the earliest applications of “curve shifters” (variables with IV characteristics) can be traced to the econometrics literature in the 1930’s [11, 12]. Indeed, PG Wright introduced IV’s for estimating the elasticities of supply and demand for flaxseed, the source of linseed oil [11, 12]. For our reassessment of the value of ecological associations, we point at the analogy between some ecological variables and IV and posit that ecological analyses are useful and meaningful when ecological variables can be interpreted as an IV *i.e.* a variable that is correlated with, or preferably a “cause of” the individual exposure, but whose association with the outcome of interest is not itself confounded, at least not by unmeasured variables (Figure 1). In addition, the instrumental variable should only affect the outcome via the individual exposure. That is, there should be no causal pathways linking IV and outcome that bypass the exposure. An epidemiological example where instrumental variables have shown to be useful is that of “Mendelian randomisation” (MR) [13]. For example, the blood pressures of individuals with alcohol aldehyde dehydrogenase deficiency mutations are compared to those without such mutations, as “on average” carriers of this mutation drink less than non-carriers [14]. Note that in this example, the use of MR also obviates the need to measure individual alcohol consumption, something that in addition to potential confounding by behavioural variables is fraught with methodological problems anyway [15]. However, MR has also been fruitfully used to evaluate a causal effect of high-density lipoprotein in blood on cardiovascular disease [16]. In addition, intention-to-treat analysis of a randomised controlled trial is also essentially based on instrumental variables, as when compliance is <100% random assignment to treatment is not identical to actual treatment received, but is not influenced by the potentially confounding factors that influence individual treatment compliance.

Noteworthy is that intention-to-treat analysis does not estimate the average causal effect among compliers and is therefore subject to “dilution” bias i.e. a bias towards the null due to misclassified exposure. Treatment allocation is typically a good instrumental variable when by using blinding there are no causal links between the randomisation arm and outcome that bypass actual treatment. In the context of clinical trials, it has been established that monotonicity is an essential requirement of treatment assignment to be a good IV. Basically, in the context of ecological studies, monotonicity means that increases at the ecological level do not lead to decreases at the individual level. For example, suppose that in heavily air polluted areas people stay indoors so often in fact that they are less exposed to particulate matters than individuals living in areas with less air pollution then there is no monotonicity. Linearity is not required, although non-linear relationships can be challenging in the analysis [17, 18]. In randomised controlled trial causal analysis terminology, this means that there are no “defiers” [19]. This idea can be simply extrapolated to other IVs. In the case of MR and alcohol consumption for example, monotonicity is plausible, as it is unlikely that anyone without the aldehyde dehydrogenase deficiency mutation would have had a higher weekly alcohol consumption in the counterfactual situation in which he/she carried the mutation. In the context of ecological associations, the IV idea can be easily translated to time series investigations when over the period of varying ecological exposure, such as air pollution or the introduction of specific legislation, the population remains the same. Monotonicity, however, could be violated if health warning about levels of environmental exposure would change an individual’s behaviour.

An appealing feature of using an ecological IV is the potential to answer research questions that may not be possible using alternative observational research designs at the individual level. For this reason, ecological studies are also popular in studies where individual observations are often hard to obtain [16]. For example, the introduction of a legal obligation to wear bicycle helmets has been explored as an IV for a study on the impact of wearing (bicycle) helmets on risk of injury. On an individual level wearing helmets is probably confounded with factors such as “carefulness” and “risk taking”. However, a legal obligation to wear helmets is unlikely to affect “carefulness” or other traffic behaviour but is an independent reason for wearing such helmets. Although the existence of defiers (people who stopped wearing helmets because of the obligation to wear one) cannot be completely ruled out they are probably rare.

Robinson [20], specifically, employed an ecological approach to explore the effectiveness of introducing a mandatory bicycle helmet law in Australia on reducing overall cyclist injury morbidity and mortality. Robinson [20] utilised population level data for injured cyclist hospital admissions and matched aggregate survey data on helmet wearing rates pre-law, first year of law and second year of law to argue that cycle helmets do not provide marked safety benefits for cyclists at the population level. Despite large increases in (mandatory) helmet use as a result of these helmet laws, the proportions of cyclists and pedestrians admitted to hospital for head injuries followed a similar, declining trend. As such, Robinson [20] attributed the observed reduction in cyclists with head injuries to the implementation of other major road safety initiatives at the same time such as the enforcement of speed limits, drink-driving laws, treatment of accident black spots, etc. Interestingly, the greatest effect of introducing the cycle helmet law appeared to discourage cycling as the pre- and post-law surveys showed reductions in numbers of child cyclists 15 and 2.2 times greater than the increase in numbers of children wearing helmets; an observation that would seem to invalidate the condition that an IV should only affect the outcome via the exposure of interest. As another example, alcohol consumption has many, often adverse, effects on health. However, on an individual level alcohol consumption is associated with many other behaviours, such as smoking and sexual risk taking, all of which may act as confounders when causally related to a health outcome of interest. Some interventions that affect alcohol consumption, such as the “natural experiment” of the 1920–1933 prohibition in the United States of America (USA), may offer opportunities to bypass this confounding and may thus act as instrumental variables. However, for this to be valid it is essential that the population could be considered (more or less) constant so that the concepts of complier and defier make sense. This latter condition would make long term time series (years, decades) questionable.

In other ecological contexts this criterion of monotonicity may not be so simple to apply; for example, when we compare regions A and B with different populations and different ecological exposure levels X. Direct comparison of an outcome measure y between A and B may not be appropriate as A and B may have different levels of other factors C that causally influence y, and are therefore confounders or effect modifiers. Another limitation of IVs is that they only can be expected to say something about the causal effect of individual exposures x_i_ on y if we assume that the relationship between x_i_ and y is monotonic. Thus, any exposure that has, say, a U-shaped relationship with outcome should preferably not be the subject of ecological analysis.

### Estimation

The existence of an ecological IV makes it possible to establish causality of the association between exposure and outcome, even when individual exposures are not observed but the IV for each individual is known. However, this is only a qualitative conclusion. In addition, it may also be desirable to estimate the strength of the relationship between individual exposures and outcome. When individual exposures are also measured this is straightforward using either two-stage least squares of residual insertion [21, 22]. In other situations this can be challenging especially in the context of non-linear relationships.

### Air pollution time series

One example in which an ecological variable would seem to have proper IV characteristics is in the case of time-varying air pollution, such as particulate matter with an aerodynamic diameter of <10 micrometres; PM_10_. In many places such concentrations vary considerably, and often rapidly (within hours or days), over time. Although measured average levels may not accurately reflect individual exposures, it seems plausible that there exist close correlations and that therefore the ecological associations can be used to study the association between air pollution and (say) episodes of asthma exacerbation. Plausible confounders, for instance temperature, are measurable and can therefore be adjusted for. Also, since largely the same individuals are observed during different time periods, the concept of defier is easy to interpret, *viz.* someone who has lower individual exposure during times of high environmental levels. Of course, such studies would seem less appropriate for outcomes that are the result of chronic cumulative exposures e.g. cardiopulmonary- or cancer-related mortality such as in the American Cancer Society Study [23].

### Geographical comparisons

More difficult is the ecological comparison between geographical areas, for example two areas with largely different air pollution levels (e.g. rural agricultural region versus an urban industrial zone). As the two populations are different, the concept of “defier” only has a counterfactual interpretation, *viz.* as someone, if he had happened to have resided in the area with the higher pollution levels would – personally, have lower exposure to air pollution (or *vice versa*). Such (counterfactual) individuals are probably rare if the areas are similar in other (relevant) aspects. However, this may not always be the case. Individuals with asthma, for example, may choose not to live in areas with high air pollution levels and are thus defiers. There is no gold standard test to assess whether geographically defined environmental exposures have IV properties but this has to be assessed on the basis of substantive knowledge. Mean individual exposures may sometimes be plausible as IVs but sometimes not.

### Poverty and health

A similar situation may exist in the widely discussed relationship between “wealth”, “poverty”, “social economic status”, “employment status” and various (health) outcomes. On an individual level, there may be a strong relationship between unemployment, say, and (poor) health and mortality, suggesting that unemployment is causally related to adverse health outcomes. However, in addition to selection biases, this relationship may be confounded by many variables, which may make a causal interpretation questionable. The unemployed individuals may have lost their jobs due to poorer physical and/or mental health or may be less socially adaptable. An economic crisis or depression with rapid massive increases in unemployment may act as an IV, and if (specific) mortality or morbidity does not increase during such depressions then one may question causal interpretations of associations on an individualistic level. Again, since largely the same individuals are involved, it is (at least conceptually) easy to assess whether there are (significant numbers of) defiers, specifically people who economically benefit (or find employment) during times of depression. Although such individuals may exist, they are probably rare. Economic depressions may also potentially affect health through pathways not involving unemployment. For example, during depressions the (still) employed may avoid behaviours that would put their employment in jeopardy. Although these alternate causal pathways could potentially invalidate the value of depressions as an IV their importance would seem minor. Similarly, natural disasters or other “natural experiments” may similarly occasionally provide good instrumental variables. A case in point is the widely accepted association between tuberculosis and poverty [24–27]; while almost all cross-sectional studies show this association the rapid increase in poverty in the USA and Europe during the 1930’s did not give rise to increases in tuberculosis mortality [28].

As a further example of the use of IV in intervention, rather than observational, studies, we consider the association between regional *s*ex worker interventions and human immunodeficiency virus (HIV) prevalence in India. This is to illustrate situations in which ecological (geographical) levels of a variable may have suitable IV characteristics. In the four high HIV-prevalence states (Andhra Pradesh, Maharashtra, Karnataka, Tamil Nadu) of Southern India interventions targeting sex workers (e.g. condom use, sexually transmitted disease treatment) are implemented on a district level. Effects of the interventions on district-level HIV prevalence and incidence are also measured on a district level by the district-specific HIV prevalence in young (<25 years) ante-natal care attendees. The idea behind these interventions is that prevention of transmission (by safer sex practices) between sex workers and their clients would reduce overall HIV transmission. These interventions received strong support from the Bill and Melinda Gates Foundation in the form of the “Avahan” project [29]. The existence of defiers, i.e. people with a lower risk of HIV infection in the absence of an intervention, is unlikely, thus monotonicity seems plausible. So in principle, intervention at a district level could be a good IV.

## Conclusion

Researchers should be reminded that no perfect epidemiological study exists and all research designs have both strengths and weaknesses. Hence, the selection of an appropriate research design requires deep consideration of the design components, research question and ability to estimate or quantify important exposure variables related to the outcome of interest. While ecological associations have an infamous reputation in epidemiology and differences between individualistic and ecological associations are often attributed to the “ecological fallacy” we have argued that this is incorrect in situations in which ecological or aggregate exposures can serve as an instrumental variable and associations between individual exposure and outcome are likely to be confounded by unmeasured variables. Thus, instrumental variables, and by consequence some ecological studies, have the advantage of reducing bias. We believe that this paper delineates situations in which ecological associations may provide reliable causal interpretations and thus provides a yardstick for assessing whether the ecological fallacy can be expected. For assessment of whether a causal relationship exists, neither individual studies, nor ecological (IV) studies by themselves suffice. Even experimental studies, often inadequately mimicking “wild type” exposures, do not suffice. Deep conclusions often require synthesis of evidence of different kinds [30]. It is our opinion that proper ecological studies can contribute to this evidence.

## Abbreviations

IV: Instrumental variable
HIV: Human immunodeficiency virus
MR: Mendelian randomisation
PM10: Particulate matter with an aerodynamic diameter of <10 Micrometres
USA: United States of America.
